# Supplementary material for: Phenoxyalkylimidazoles with an oxadiazole moiety are subject to efflux in Mycobacterium tuberculosis
Source: PLoS One. 2021 Jan 22;16(1):e0239353. doi: 10.1371/journal.pone.0239353 (PMC7822546; doi:10.1371/journal.pone.0239353)
Supplement: Attachment — Submitted filename: Rebuttal.docx [file pone.0239353.s001.docx]

**Reviewer #1:**

In previous publications, the antituberculosis activity of PAI compounds has been fully described, and now, in the present manuscript, the impact of efflux on their activity has been investigated.

In this work, it is reported that verapamil, a widely used and very potent efflux inhibitor in mycobacteria, has failed to decrease MICs of the PAI compounds. A potential explanation could be that in this work, verapamil is used at 20 µM whereas in most publications on the field verapamil is used at 180 µM (or even higher concentrations!), without compromising cell viability. Maybe, such a difference in concentration could explain this discrepancy. It is strongly suggested to further test this concentration experimentally.

>As noted in the manuscript, we used the highest concentration of verapamil at which growth was unaffected in our assays. Concentrations higher than 20 µM resulted in growth inhibition with our strain and in our assay. Since higher concentrations affected growth, we conclude that 20 µM should have an effect on efflux. We cannot comment on how other groups were able to use verapamil at higher concentrations without compromising growth. From the manuscript:

“We first determined the concentration of efflux inhibitors to use by running a dose response and selecting the highest concentration at which *M. tuberculosis* growth was not affected: 12.5 μM for reserpine, 20 μM for verapamil and 2.5 μM for carbonyl cyanide m-chlorophenylhydrazine (CCCP).”

The relationship between presence of oxadiazole moieties and efflux susceptibility has been explored further by analysing a few other compounds. First, structures shown in Figure 1 could maybe be arranged in a different way in order to make easier the identification of structural similarities and differences between them. Second, I agree with the authors’ perception (shown in the last paragraph of the manuscript) that a much wider number of compounds should be assayed in order to fully support (or not) the influence of this chemical part in efflux susceptibility.

>We have revised Figure 1.

>We agree that a thorough study of a wide range of chemical moieties might be able to determine the determinants of efflux, but we feel this is beyond the scope of this manuscript. We have clarified and expanded our rationale for conducting this study. The data generated are important, not for understanding efflux per se, but for guidance in a drug discovery program looking at structure-activity relationships using whole cell activity as the only driver of medicinal chemistry. We have demonstrated that efflux plays a small, but significant role in the activity of two compounds from the PAI series. We have included data from four additional compounds with the diazole moiety which were not subject to efflux, which strengthens our conclusion (Table 1). These data suggest that future development of this series should also consider whether compounds are subject to efflux, especially when looking at structure-activity-relationships. The conclusions are relevant for drug discovery and compound progression. We have tempered and clarified our conclusions to reflect this.

Minor points:
Typos: In methods section, albumen -> albumin; second line in MIC paragraph;

>Corrected
First four lines in Results and Discussion could be moved to introduction

> We have moved as suggested.
Description of MIC protocol is duplicated in Methods and in Results and Discussion.

>We have deleted from the results section.
It would be good to read a brief description of the activity of QcrB protein.

>We have added this.

**Reviewer #2:**

This work examines the effect of efflux inhibitors on a couple of compounds. Two of the compounds have an oxadiazole belonging to the PAI series, one an oxazole and one is an indole-containing PAI compound. Some control compounds are included. The efflux inhibitors have different effects on the MIC of the compounds. The MIC of two compounds (351551 and 105909) are affected by a Rv0678 mutation although a transposon mutant in mmpL5 shows no difference in MIC. From the limited series of compounds explored it seems hard to make structure-associated conclusions about similarities in efflux although reserpine affects the MIC of several compounds.

**Major comments**
A much broader series of compounds need to be explored if any conclusions are to be made regarding the effect of a chemical moiety on efflux.

>See point for reviewer 1. We have clarified the rationale for this study as supporting a drug discovery effort and included additional data.

Minor comments
The inclusion of MIC testing against the Rv0678 mutant strains and mmpL5 inactivation strain adds data but little clarity. The mutations in Rv0678 confer a small increase in resistance whereas inactivation of mmpL5 gives very little difference overall for most compounds to the MIC compared to the parental strain. For example, for 351551 the Rv0678 mutant has a 2-fold higher MIC but the mmpL5 Tn mutant is not more sensitive than its parental strain. The other compounds are not affected by the Rv0678 mutation.
From just two compounds containing an oxadiazole, it seems hard to make any substantial conclusion about similarities in efflux. The MIC of one is affected by the Rv0678 mutation and the other one hardly at all. Reserpine affects the MIC of both but it also affects the MIC of the oxazole and the non-oxadiazole-containing PAI compound - it may be less than 2-fold, but all the differences are modest yet significant.

>We agree that the differences are small, but the data are robust. We think the information is important to present in the publication but can move to supplementary information if required.
