# Supplementary material for: Phenoxyalkylimidazoles with an oxadiazole moiety are subject to efflux in Mycobacterium tuberculosis
Source: PLoS One. 2021 Jan 22;16(1):e0239353. doi: 10.1371/journal.pone.0239353 (PMC7822546; doi:10.1371/journal.pone.0239353)
Supplement: Attachment — Submitted filename: Rebuttal.docx [file pone.0239353.s002.docx]

**Reviewer #1:**

As noted by the reviewer, the concentration of verapamil we used was lower than in other studies. We were cautious in making sure there was no inhibition of growth at all (%inhibition ,20) and we had seen variability between batches from suppliers. We have removed the conclusion from the abstract and added a caveat regarding this low concentration and that it may not be sufficient to inhibit efflux.
